# Health inequities in pediatric anaphylaxis: A policy framework and call to action for equitable care from SIAIP allergy prevention commission

**DOI:** 10.1111/pai.70394

**Published:** 2026-06-10

**Authors:** Carolina Grella, Angela Klain, Amelia Licari, Sara Manti, Francesca Mori, Francesca Galletta, Antonio Andrea Senatore, Leonardo Tomei, Michele Miraglia del Giudice, Gian Luigi Marseglia, Cristiana Indolfi, The SIAIP Primary and Secondary Prevention of Allergic Diseases Commission

**Affiliations:** ^1^ Department of Woman, Child and General and Specialized Surgery University of Campania ‘Luigi Vanvitelli’ Naples Italy; ^2^ Pediatric Unit, Department of Clinical, Surgical, Diagnostic and Pediatric Sciences University of Pavia Pavia Italy; ^3^ Pediatric Clinic, Fondazione IRCCS Policlinico Pavia Italy; ^4^ Pediatric Unit, Department of Human Pathology in Adult and Developmental Age ‘Gaetano Barresi’ University of Messina Messina Italy; ^5^ Allergy Unit Meyer Children's Hospital IRCCS Florence Italy

**Keywords:** anaphylaxis, children, disparities, epinephrine autoinjector, prevention, recommendations

## Abstract

Pediatric anaphylaxis is a rapidly evolving and potentially fatal allergic reaction whose incidence is increasing worldwide, particularly among young children. Despite international guidelines identifying intramuscular epinephrine as the first‐line, life‐saving treatment, significant health inequities persist in the prevention, recognition, and management of pediatric anaphylaxis. This manuscript, developed by the Primary and Secondary Prevention of Allergic Diseases Committee of the Italian Society of Pediatric Allergy and Immunology (SIAIP), moves beyond a traditional narrative review by integrating current evidence with a structured policy‐oriented framework. Evidence highlights pronounced socioeconomic, racial, cultural, biological, and geographic inequities influencing disease burden, access to specialist care, prescription and availability of epinephrine autoinjectors (EAIs), training of caregivers and healthcare professionals, and timely epinephrine use in emergency settings. Building on this evidence, we propose a SIAIP‐driven call to action and outline a preliminary national policy framework aimed at reducing inequities through coordinated interventions. Key priorities include equitable access to EAIs, standardized and repeated practical training, school‐based preparedness programs, public awareness initiatives, integration of psychological support, and the development of national monitoring systems. By translating evidence into actionable strategies, this work provides a national perspective that complements existing international recommendations and offers a model for other countries seeking to address disparities in pediatric anaphylaxis care.

**Key message**

This work highlights how health inequities across socioeconomic, geographic, educational, and policy domains significantly affect the prevention and management of pediatric anaphylaxis. This work integrates current evidence with a structured, equity‐oriented action framework developed by the Primary and Secondary Prevention of Allergic Diseases Committee of the SIAIP. It provides clinicians, policymakers, and educators with practical and measurable strategies to reduce disparities, improve equitable access to epinephrine, and strengthen preparedness across healthcare and community settings. The proposed action plan also provides a national model that may inform the development of similar initiatives by other national scientific societies and within different healthcare systems.

## 1 INTRODUCTION TO PEDIATRIC ANAPHYLAXIS

Pediatric anaphylaxis is a sudden, potentially life‐threatening allergic reaction requiring immediate recognition and management. In the United States, longitudinal data from 2001 to 2010 show a 4.3% annual increase in anaphylaxis incidence, with the highest rise among children aged 0–9 years.^1^ Clinically, anaphylaxis presents with respiratory distress, cardiovascular compromise, and cutaneous manifestations, which can progress rapidly. International societies provide consistent definitions: the European Academy of Allergy and Clinical Immunology (EAACI) defines it as a serious, rapid‐onset reaction that affects multiple organ systems and frequently requires urgent medical intervention, with respiratory and/or cardiovascular involvement often accompanied by skin or mucosal symptoms^2^; the World Allergy Organization (WAO) describes it as a severe systemic hypersensitivity reaction with airway, breathing, or circulatory compromise; and the American Academy Allergy Asthma & Immunology (AAAAI) emphasizes multi‐system involvement or severe dysfunction of a single organ.^2^ Early symptoms, often cutaneous such as urticaria or angioedema, may precede other organ involvement and can be subtle in children, requiring high clinical suspicion.^2^ Common triggers include foods, drugs, and hymenoptera venom, with food allergens being the predominant cause in childhood. Peanuts, tree nuts, cow's milk, hen's egg, shellfish, and wheat are most frequent, though regional, cultural, and dietary factors influence prevalence.^3^, ^4^, ^5^, ^6^ Prompt recognition and immediate intramuscular epinephrine (IM epinephrine) remain the cornerstone of management.^7^, ^8^, ^9^ Epinephrine is a non‐selective α/β adrenergic agonist: α₁ induces vasoconstriction to counter hypotension, β₁ increases cardiac output, and β₂ promotes bronchodilation and reduces vascular permeability, stabilizing multiple organ systems during anaphylaxis.^10^ Current recommendations advise 0.15 mg autoinjectors for children under ~25–30 kg and 0.30 mg for heavier children, with adjustments for specific clinical scenarios.^11^ Autoinjectors are the preferred delivery method outside medical settings, being portable, prefilled, and ready to use. Proper training and availability of two doses are central to effective intervention. Recently, intranasal epinephrine has emerged as a needle‐free alternative, FDA‐approved in 2024 for patients ≥30 kg and extended in 2025 to 15–30 kg children.^12^, ^13^ The pre‐measured spray allows rapid administration, reduces injection errors, and is suitable for caregivers or non‐medical personnel, though current guidelines do not yet recommend it as a full replacement for IM injection. Adjunctive therapies, including oxygen, fluids, antihistamines, and corticosteroids, remain supportive and must not delay epinephrine.^14^ Long‐term prevention is essential to reduce recurrence. Strategies include allergen avoidance, education of patients, caregivers, and school personnel, correct use of epinephrine autoinjectors (EAI), and in select cases, allergen immunotherapy (AIT), particularly for hymenoptera venom and increasingly for food allergens under specialist supervision.^15^ Prevention requires integrated healthcare systems that ensure timely access to diagnosis, treatment, and education, minimizing socioeconomic and regional inequities. Precision medicine and endotype‐driven approaches underscore the need for equitable access to specialized allergy services across pediatric populations.^16^

**FIGURE 1:**
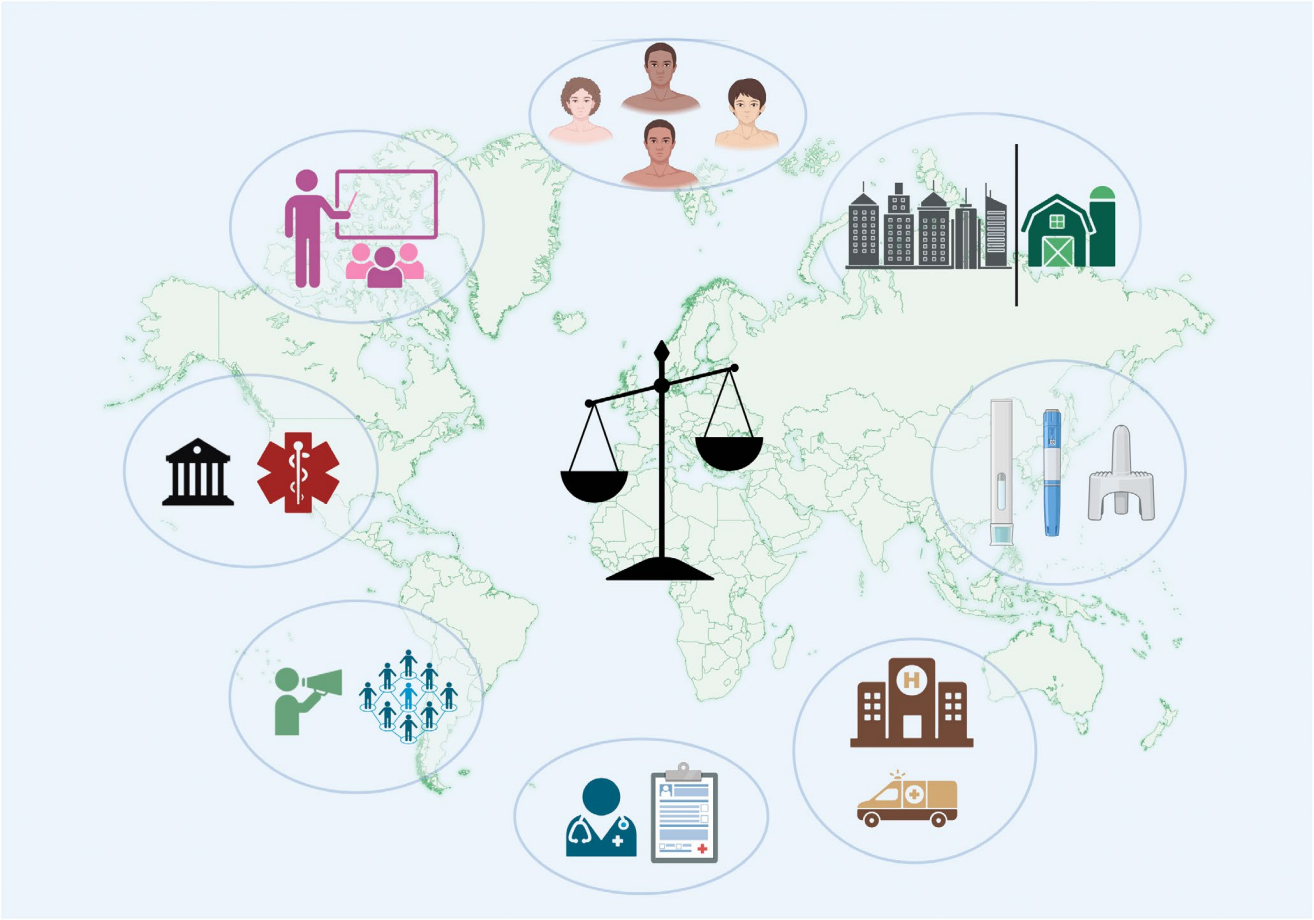
The central balance symbol represents disparities in access to diagnosis, treatment, and emergency management across different populations and settings. Surrounding icon clusters illustrate key domains contributing to inequities, with a clear visual correspondence: The group of faces at the top represents demographic and biological factors (age, sex, and ethnicity); the teacher with students (top left) represents educational factors, including training of caregivers, school personnel, and healthcare providers; the urban–rural landscape (top right) represents socioeconomic and environmental context (urban–rural differences and living conditions); the medical devices on the right represent access to epinephrine autoinjectors and other medical devices; the hospital and ambulance (bottom right) represent healthcare system organization and access to services (availability of specialists, emergency care, and referral pathways); the clinician with medical chart (bottom center) represents clinical management and diagnosis processes; the megaphone with a network of people (bottom left) represents community awareness and communication; and the institutional building with medical symbol (left) represents public health policies and reimbursement frameworks. These interconnected determinants influence preparedness, timely recognition, and treatment of anaphylaxis, ultimately leading to unequal outcomes across regions and populations. Created with BioRender.com.

This manuscript highlights existing health inequities in pediatric anaphylaxis prevention and management. Diagnostic challenges, variability in clinical presentation, and disparities in access to care exacerbate delays in recognition and treatment. Inclusive health policies should promote equitable access to early diagnosis, education, emergency care, and long‐term management strategies to ensure safe and effective outcomes for all children at risk of anaphylaxis Figure 1.

## 2 LITERATURE SEARCH STRATEGY

This narrative review was based on a targeted literature search conducted in PubMed and Web of Science. The search focused on pediatric anaphylaxis, epinephrine use, epinephrine autoinjectors, health inequities, disparities in access to allergy care, reimbursement policies, educational interventions, and school‐based preparedness. Priority was given to recent clinically relevant studies, international guidelines, position papers, and policy‐oriented publications. Additional references were identified through manual screening of reference lists and expert selection by the authors. Given the narrative and policy‐oriented scope of the manuscript, no formal systematic review protocol or meta‐analysis was performed.

## 3 HEALTH INEQUITIES IN PEDIATRIC ANAPHYLAXIS

Health inequities in pediatric anaphylaxis stem from the interaction of biological, social, and systemic factors affecting exposure, recognition, and access to care. These disparities reflect broader social determinants, including education, housing stability, environmental exposures, and systemic discrimination, which collectively influence health outcomes. Key domains include: biological and demographic factors such as age and sex affecting risk and disease expression^1^; socioeconomic and cultural determinants, including income, ethnicity, and health literacy^2^; geographic disparities related to unequal healthcare access and environmental exposures^3^; and educational and systemic gaps in professional and caregiver training and policy frameworks.^4^ These interconnected dimensions lead to unequal preparedness and outcomes in pediatric anaphylaxis prevention.

### 3.1 Socioeconomic, racial, and cultural inequities

Racial and ethnic minority children bear a disproportionate burden of food allergy (FA) and anaphylaxis, with higher rates of sensitization and severe reactions among Black and Hispanic populations compared with White peers.^17^ Large U.S. cohort studies confirm that non‐Hispanic Black and Hispanic children have higher odds of severe reactions, greater emergency department utilization, and lower lifetime epinephrine use, highlighting structural inequities in both prevention and acute management.^18^, ^19^ Even after adjusting for socioeconomic status, non‐White and second‐generation immigrant children show higher rates of sensitization and clinically significant allergic disease.^20^ A systematic review similarly reported that Black children have increased odds of food sensitization, multiple allergies, and comorbid asthma, yet are less likely to receive specialist care or confirmed diagnoses.^21^ The AAAAI 2025 Position Statement emphasizes delayed diagnosis, under‐prescription of epinephrine autoinjectors (EAI), and limited access to specialists as key contributors to increased morbidity and mortality among racially and ethnically minoritized pediatric populations.^22^ Collectively, these disparities arise from biological susceptibility, environmental exposures, and systemic inequities in healthcare access and preventive education.

### 3.2 Biological and demographic inequities

Age significantly influences pediatric anaphylaxis expression and severity. Older children and adolescents are more prone to severe reactions, likely reflecting cumulative allergen exposure, physiological maturation, and risk‐taking behavior.^4^ Turkish multicenter data show mild reactions predominate in infants, with moderate‐to‐severe episodes and cardiovascular involvement increasing with age.^23^ Similarly, a Thai cohort demonstrated a higher likelihood of severe outcomes in adolescents, whereas infants had shorter reaction times but milder symptoms.^24^ Symptom patterns also differ developmentally: infants often present with mucocutaneous or neurologic signs, whereas older preschoolers exhibit more respiratory symptoms.^25^ Sex influences prevalence, severity, and clinical expression. Boys predominate in early childhood food‐induced anaphylaxis, while adolescent girls show increased severity and higher rates of drug‐ and venom‐induced reactions.^26^, ^27^ Data from Türkiye indicate males account for 51.3% of emergency admissions, but females experience higher ICU admissions and fatal outcomes.^28^ Mechanistically, estrogens enhance mast cell activation, increasing susceptibility, whereas androgens have immunosuppressive effects, explaining male predominance early in life but lower severity in adulthood.^23^, ^26^, ^27^, ^28^ Female‐specific phenotypes, including catamenial and hormone‐related anaphylaxis, underscore the need for sex‐specific diagnostic and therapeutic strategies.^28^

### 3.3 Geographic inequities in access to care

Geographic variation represents a crucial determinant of inequities in pediatric anaphylaxis outcomes, with consistent evidence showing that children residing in rural or underserved regions experience higher morbidity, delayed treatment, and reduced access to specialist care.^29^ These data are highlighted in numerous literature studies. In a Turkish national cohort, the highest fatal anaphylaxis rates were reported in rural and mountainous regions such as the Black Sea and Central Anatolia, primarily due to delayed epinephrine administration, limited access to allergists, and insufficient emergency response capacity.^28^ Comparable patterns have been observed across Europe, where rural‐residing children face greater barriers to allergy diagnosis, immunotherapy, and access to EAI, resulting in poorer anaphylaxis management and increased mortality risk.^29^ Recent multicenter studies underscore that geographic location directly influences both the severity and timeliness of anaphylaxis management, with rural children less likely to receive prompt IM epinephrine or follow‐up allergy evaluation. In the Thai pediatric cohort of 335 patients, severe anaphylaxis was significantly associated with older age (>12 years), cardiovascular manifestations, and rapid symptom onset, but also with delays in emergency care presentation, particularly in children residing outside urban centers, highlighting the interplay between geography and access to acute management.^30^ Population‐based analyses in the U.S. reveal that children residing in urban areas have nearly threefold higher rates of emergency department visits for food‐induced anaphylaxis compared with rural peers, with incidence rates of 12.31 versus 4.60 per 100,000 children, respectively.^31^ Complementary findings from Illinois indicate that urban centers such as Chicago report the highest hospitalization and emergency department visit rates for food‐induced anaphylaxis nationwide, further linking urban density, socioeconomic disadvantage, and allergic disease burden.^32^ Evidence from the English National Health Service further supports this association, revealing that individuals from socioeconomically deprived wards travel significantly shorter distances for hospital care, independent of hospital proximity, while rural residents face substantially longer travel times, particularly for emergency admissions. The study also highlights that patients from affluent areas are more likely to exercise healthcare choice and access specialized centers, whereas families in deprived or rural wards face transport and informational barriers that restrict timely care, reflecting broader structural inequities in healthcare distribution.^33^ Conversely, in high‐income Asian settings, rural regions exhibit a paradoxically higher prevalence of anaphylaxis but lower rates of EAI prescription, reflecting deficiencies in healthcare access and medication distribution networks. In Korea, the prevalence of anaphylaxis reached 28.8 per 100,000 in rural areas versus 17.3 in urban areas, yet EAI prescription rates were four times lower in rural patients (3.1% vs. 12%).^34^ From a global standpoint, urbanization and healthcare density correlate with improved access to specialized allergy services, yet rural regions across Asia and Europe continue to report lower rates of pre‐hospital epinephrine use and higher ICU admissions, reflecting systemic inequities in resource distribution.^28^, ^29^ In the Asia‐Pacific region, rural healthcare systems frequently lack essential medications such as EAI, and logistical constraints further delay emergency response, emphasizing the need for national policies ensuring equitable drug availability.^35^ Evidence from large‐scale population analyses demonstrates that the distribution of allergy specialists is markedly skewed toward urban, high‐income regions, leaving rural and low‐resource communities with limited diagnostic and preventive services. A national survey revealed that only 55.5% of allergists in the United States accept Medicaid, with state‐level acceptance rates varying from 13% to 90%, effectively excluding many children in rural or economically disadvantaged regions from specialty care. The Center for Food Allergy and Asthma Research (CFAAR) identified geographic maldistribution of allergists as one of the leading systemic drivers of inequity, emphasizing the importance of mobile health clinics (MHCs), telemedicine, and community‐based outreach models to bridge rural access gaps. In fact, mobile health units delivering free allergy and asthma care, such as the Mobile Care Chicago model, have successfully reduced emergency department visits to 4% among enrolled children, demonstrating the potential of decentralized care systems to mitigate geographic barriers. Beyond infrastructure, geographic inequities intersect with broader social determinants of health (SDoH), including neighborhood deprivation, housing quality, environmental exposures, and transportation barriers. Families living in high‐deprivation index (ADI) areas encounter higher allergen loads, increased environmental pollutants, and reduced access to allergen‐free foods, collectively contributing to a disproportionate burden of allergic emergencies. Environmental injustice remains a major driver of regional disparities: children residing in historically redlined or industrial neighborhoods face higher exposure to air pollutants, mold, and allergens, factors linked to increased rates of asthma and anaphylaxis‐related hospitalizations.^36^

### 3.4 Educational and systemic inequities

Training and proficiency in EAI use remain inconsistent across healthcare and community settings, creating inequities in pediatric anaphylaxis preparedness and outcomes. Studies consistently show that both caregivers and professionals often lack the skills and confidence to administer epinephrine correctly. In the UK, a prospective study of 122 children with FAs revealed that 69% of parents could not use an EpiPen correctly, did not have it available, or were unsure when to administer it. Parents who received a prior practical demonstration were four to five times more likely to use the device correctly, and those who consulted allergy specialists or national self‐help organizations were four to six times more competent.^37^ Similarly, a French study evaluating 111 children prescribed Anapen® found suboptimal parental preparedness. Although 90% received demonstrations, only 76% practised with a training device, fewer than half received written instructions, and among school‐aged children, only 54% had a personalized care plan and 60% had a complete emergency kit at school.^38^ Parental anxiety remains a major barrier: in the UAE, despite 94% receiving in‐person EAI training, only 36% felt confident, with fear of causing harm delaying or preventing administration during actual anaphylaxis episodes.^39^ Healthcare providers also demonstrate deficiencies. Only 25% of medical professionals correctly performed all essential steps, and over 75% were unaware of available dose strengths.^40^ In a controlled study of 160 physician trainees, correct demonstration rates declined substantially within three to 6 months, highlighting the need for recurrent training.^41^ Errors are common, particularly premature device withdrawal before the recommended 10‐s hold. Teachers and school staff, often first responders, also show low competence. In Ireland, only 10% of primary school staff performed all steps correctly, and self‐reported confidence did not predict actual performance, underscoring inadequacies in current educational programs.^42^ Digital resources present further challenges: among 107 English‐language YouTube videos, only those produced by healthcare professionals met quality and reliability standards, while 18% of user‐generated videos contained misleading or harmful information.^43^ Emerging interventions emphasize psychologically informed approaches to improve adherence and reduce fear. A UK interventional study demonstrated that a 90‐min workshop incorporating behavioral and motivational techniques improved clinician knowledge, confidence, and likelihood of teaching others, with effects persisting 6–8 weeks post‐training.^44^ Overall, disparities in EAI training reflect systemic and psychosocial inequities. Inconsistent, infrequent, and context‐insensitive education undermines emergency readiness.

### 3.5 Disparities in prescription

In Japan, a nationwide population‐based analysis using the National Health Insurance Claims Database reported 88,039 individuals prescribed EAIs during a one‐year period, corresponding to 69.5 prescriptions per 100,000 population‐years. Children aged 0–9 years had the highest prescription rate (278.9 per 100,000), with children under 19 years being 6.4 times more likely to receive an EAI than adults, with the highest rate among those aged 0–9 years (278.9/100,000 population‐years). However, the average number of devices per patient was only 1.33 per year, lower than in the U.S. or UK, where dual‐prescription practices are standard.^45^ It should be noted that the indicators used across studies are not fully comparable, as the Japanese data are expressed both as population‐based prescription rates and average devices per patient, whereas UK and U.S. studies often report prescriptions per high‐risk patient or per person‐years, which may partially explain differences in reported prescribing intensity. A national retrospective cohort study from the UK analyzed data from over one million children in primary care between 2000 and 2012, revealing a 355% increase in the number of children prescribed EAIs and a 506% increase in the total number of devices issued per 1000 person‐years. On average, each high‐risk child received 3.8 devices per year, with nearly half prescribed four or more devices annually. Despite this rise, only about 50% of prescriptions were associated with a documented allergy or anaphylaxis diagnosis, raising concerns about prescribing rationale and cost‐effectiveness.^46^

### 3.6 Availability of epinephrine and policy differences in access and reimbursement

The WAO global survey conducted in 2023 by the WAO Anaphylaxis Committee across 66 countries showed that EAIs are available in only 60% of countries, with access concentrated in high‐income regions, while many low‐ and middle‐income nations, particularly in South America, Africa/Middle East, and Asia–Pacific, either lack EAIs entirely or depend on importation or “named‐patient” licenses, leading to unstable supply chains and inequitable access. Only 16% of countries reported having national policies ensuring EAI availability in public spaces such as schools, public transport, or parks, highlighting the limited integration of anaphylaxis preparedness into public health infrastructure. Regional differences are substantial: in Europe, 43.5% of countries manufacture EAIs locally and 47.8% rely on importation, whereas in North America, multiple devices (Anapen®, Auvi‐Q®, Epipen®) are nationally produced and marketed; by contrast, in the Africa/Middle East region, 90% of surveyed countries reported no national manufacturing or importation capacity, underscoring severe structural barriers to access.^47^ Where EAIs are unavailable, physicians may dispense adrenaline ampoules with syringes or prefilled syringes as alternatives, although these are associated with dosing inaccuracies, sterility issues, and instability due to temperature fluctuations.^48^ ^,^ ^49^ According to the WAO global update published in 2009, the median international cost of a single EAI was US$97.87 (range US$54.50–168.66), a level disproportionately high relative to average household income in many low‐ and middle‐income countries. That same report showed that only 27% of surveyed countries offered any financial assistance or insurance reimbursement for EAIs, while partial reimbursement was available in Australia, Canada, Italy, Portugal, and the UK, where EAIs were fully subsidized for children under 16 years of age.^49^ Recent WAO data confirm that EpiPen® costs in Europe are up to ten times lower than in the U.S., even when imported, illustrating the persistent price disparity between markets.^47^ A complementary global analysis of national essential medicines lists (NEMLs) further demonstrated wide heterogeneity in the inclusion of adrenaline and other essential drugs; of 137 countries studied, only 70% had up‐to‐date NEMLs, and discrepancies with the WHO Model List were substantial and only partly explained by income level or health expenditure.^50^ In Ethiopia, even when adrenaline was listed as an essential medicine, availability in health facilities reached only 36.7%, and nearly 80% of surveyed essential drugs were unaffordable, reflecting major procurement and reimbursement deficiencies.^51^ Globally, 81% of countries require a medical prescription for EAIs, while a minority, including Australia, Canada, Greece, Israel, and Thailand, permit non‐prescription or “behind‐the‐counter” sales under controlled conditions.^49^ In countries without official distribution channels, EAIs may be accessed via named‐patient importation or informal “suitcase trade”, the latter posing risks of supply interruption and degradation from temperature excursions during transport or storage.^47^ Low‐ and middle‐income countries remain disproportionately affected by lack of national manufacture, absence of reimbursement frameworks, and dependence on importation, while even in regions with formal access, high prices, limited shelf life (typically 12–18 months), and inconsistent policy support undermine equitable prevention and readiness.^47^, ^49^

Moreover, although epinephrine is classified by the WHO as an essential medicine, EAIs are absent from most national essential medicines lists, revealing a critical gap between international recommendations and national implementation^50^ (Table 1).

**TABLE 1:** Availability of EAIs by region, showing the proportion of countries with national manufacture, import‐only supply, no availability, and the presence of public‐setting policies.

State	EAI national manufacture (%)	Import‐only (%)	No availability (%)	Policy in public settings (%)
North America	100	0	0	50
Europe	43.5	47.8	8.7	78.3
South America	0	27.8	72.2	0
Africa/Middle East	0	10	90	0
Asia‐Pacific	17.6	41.2	41.2	23.5

*Source:* From Tanno et al.,^47^ *World Allergy Organ J*. 2023, data.

In the Italian context, recent national data further confirm the presence of regional disparities in the availability and reimbursement of epinephrine autoinjectors. A nationwide survey conducted by SIAIP and AAIITO revealed that certain regions, such as Sardinia and the Autonomous Province of Bolzano, reimburse only one autoinjector annually, while in others, including Piemonte, Veneto, and Valle d'Aosta, the provision of a second device is conditional and restricted to specific clinical circumstances, such as severe or near‐fatal anaphylaxis, biphasic reactions, mastocytosis, or particular logistic factors (e.g., remote residence), resulting in non‐uniform access pathways. Although pediatric access appears relatively more homogeneous compared with adult populations, as most regions reimburse two devices per year, relevant variability persists across the country. These differences, largely driven by the absence of harmonized and updated regional policies, highlight persistent structural inequities that may impact preparedness and timely access to life‐saving treatment even within the pediatric population.^52^ Although the Italian National Health Service provides coverage for these devices, the lack of a unified national reimbursement framework results in heterogeneous regional implementation.

### 3.7 Healthcare access and utilization patterns

Disparities in healthcare access and utilization substantially influence the prevention and management of pediatric anaphylaxis. Children from minority groups are less frequently referred to allergy specialists and receive EAI. Insurance coverage is a key determinant: those insured through Medicaid or lacking insurance encounter systemic barriers to specialist care and essential medications compared with privately insured peers. In addition, publicly funded healthcare systems may be insufficiently resourced to deliver comprehensive allergy services in underserved areas.^53^, ^54^

### 3.8 Underuse in emergency settings

Despite clear international recommendations identifying IM epinephrine as the first‐line and life‐saving treatment for anaphylaxis, multiple studies consistently demonstrate significant underuse in pediatric emergency departments worldwide. In a large French cohort, only 32.7% of children received epinephrine, either pre‐hospital or in the emergency department, with physicians often reserving administration for the most severe (grade III) cases, reflecting an “intuitive” but inappropriate adaptation to severity.^55^ Comparable findings have been reported in the U.S., where only 47% of children diagnosed with anaphylaxis received epinephrine in the emergency setting, and a majority were treated instead with antihistamines or corticosteroids, despite their delayed and limited efficacy.^56^ Earlier data from Russell and colleagues also found IM epinephrine use in just 56% of pediatric cases, and only one‐third of these patients received allergy referrals at discharge, underscoring persistent gaps in both acute and follow‐up management.^57^ Underuse is not limited to Western settings. A population‐based Korean study revealed that less than half of pediatric patients with anaphylaxis received epinephrine in the emergency department, highlighting similar global trends of delayed or absent treatment.^58^ The 20‐year Italian analysis by De Filippo et al. demonstrated that fewer than 60% of children with anaphylaxis received epinephrine, and only one in five received it before hospital arrival, confirming that underuse persists even in high‐income settings.^59^ Several structural and behavioral barriers contribute to this phenomenon. Fear of adverse cardiovascular effects, diagnostic uncertainty, and reliance on adjunctive medications persist among clinicians, even in tertiary centers. Furthermore, EAI prescriptions upon discharge remain suboptimal, reported in only 31–63% of cases, limiting future preparedness.^60^

## 4 GLOBAL INITIATIVES ON ANAPHYLAXIS BY INTERNATIONAL SOCIETIES

International organizations and scientific societies have increasingly emphasized the need for coordinated strategies to reduce disparities in the prevention and management of anaphylaxis, promoting public awareness campaigns, policy initiatives, and harmonized prescribing practices across regions. These efforts underscore the urgency of ensuring uniform territorial access to EAI and implementing public education programs capable of improving anaphylaxis recognition and reducing treatment delays.^61^ Among these initiatives, a particularly significant contribution comes from the *Adverse Reactions to Foods Committee* of the AAAAI, which formally supports the mandatory introduction of stock epinephrine in all schools, accompanied by standardized training for school staff, in order to ensure a rapid and equitable emergency response, independent of individual prescriptions. The report highlights that many severe pediatric anaphylactic reactions occur at school, often in children without a known allergy diagnosis or without a prescription for an autoinjector, making non‐student‐specific epinephrine essential and accessible to the entire school population. The main barriers identified include a lack of legal protection for staff who administer epinephrine, the high cost of autoinjectors, and the shortage of trained nurses or personnel. The proposed measures include legislation requiring stock epinephrine in all schools, ongoing training programs, reimbursement or subsidy policies to reduce device costs, and broad information campaigns to raise awareness and acceptance among families, schools, and communities.^62^ These efforts are complemented by numerous international awareness campaigns and educational programs aimed at improving knowledge and preparedness at both professional and community levels. The EAACI promotes *Anaphylaxis Awareness Day* on the 21st of November, a global initiative dedicated to increasing awareness on the recognition, prevention, and management of anaphylaxis, with educational materials, outreach campaigns, and multimedia content emphasizing the importance of timely intervention and equitable access to life‐saving treatments.^63^

In the UK, Anaphylaxis UK provides support, training, and advocacy for patients, caregivers, schools, and healthcare professionals, promoting initiatives for prevention, safety, and access to epinephrine.^64^ Similarly, in Australia, the Australasian Society of Clinical Immunology and Allergy (ASCIA) has developed standardized *Action Plans for Anaphylaxis*, customizable documents for managing anaphylactic emergencies, widely used in schools and healthcare settings.^65^ Digital education represents a further strategic tool: the AAAAI has developed an *Interactive Anaphylaxis Learning Guide*, a web‐based interactive module using real‐life scenarios, allowing school staff, caregivers, and healthcare professionals to practice early recognition and effective management of anaphylaxis.^66^ In Italy, similar initiatives are promoted by the Italian Society of Allergy, Asthma and Clinical Immunology (SIAAIC), which has organized institutional events and thematic conferences dedicated to anaphylaxis, such as the June 5, 2025 meeting in Rome on prevention and awareness, and the November 24, 2025, conference focused on clinical updates and patient safety policies.^67^ AAIITO promotes *World Allergy Week 2025* and the *Global Food Allergy and Anaphylaxis Forum* (*GA* ^*2*^ *FA 2025*), international forums dedicated to education, scientific exchange, and the development of strategies to reduce disparities in access to care and education on allergy risks.^68^ These initiatives, combining training, public awareness, advocacy, and development of practical guidelines, represent fundamental tools to ensure a coordinated global approach to anaphylaxis prevention, reducing disparities in timely access to life‐saving care for all children at risk.

To overcome disparities in access and outcomes, contemporary frameworks increasingly recommend multisectoral interventions that integrate the expansion of telemedicine, community‐based training, and policy‐driven reforms aimed at improving emergency preparedness and device accessibility in underserved settings. The CFAAR Summit specifically outlines strategic priorities such as insurance coverage for telemedicine‐based allergy care, implementation of legislation for stock epinephrine availability in public settings, and support for culturally competent educational programs in high‐risk communities.^35^ Recent analyses also warn that reductions in federal public‐health funding and environmental regulations threaten ongoing progress, disproportionately harming rural and minority populations that rely on publicly supported prevention programs and allergy education initiatives.^36^ Similar inequities have been reported in the Asia–Pacific region, where the availability of epinephrine, diagnostic services, and specialist follow‐up remains highly variable, further emphasizing the international relevance of systemic disparities.^69^

At the same time, the psychosocial aspects of chronic allergic risk, long recognized as central to pediatric well‐being, must be addressed to ensure comprehensive and equitable care. Children living under the threat of anaphylaxis often experience anxiety, hypervigilance, and reduced quality of life, making psychological support an essential component of modern allergy management.^70^

The implementation of these strategies requires careful consideration of feasibility across different healthcare settings, particularly in low‐ and middle‐income countries. The cost of epinephrine autoinjectors, their limited availability, the need for ongoing training of healthcare professionals and caregivers, and logistical challenges in device distribution represent significant barriers. Furthermore, the economic sustainability of educational programs and universal access policies must be evaluated in relation to available resources. Cost‐effective interventions, including community‐based training programs, decentralized care models, and targeted reimbursement policies, may enhance the practical implementation of these recommendations and facilitate their integration into clinical practice and health policy.

These initiatives, which integrate public awareness, education, policy reforms, telemedicine, and psychological support, represent a comprehensive global strategy to reduce disparities and ensure timely access to life‐saving care for all children at risk of anaphylaxis.

### 4.1 Future directions and research gaps

Despite advances in the understanding and management of pediatric anaphylaxis, important gaps in the literature remain. In particular, further research is needed on models of care in rural and remote areas, the effectiveness of telemedicine in allergy and anaphylaxis management, and strategies to ensure long‐term retention of skills in epinephrine administration. In addition, evidence on the large‐scale impact of educational interventions and public health policies in reducing inequities remains limited. Addressing these gaps will be essential to inform targeted strategies and improve clinical outcomes for children at risk of anaphylaxis.

## 5 ACTION FRAMEWORK FOR REDUCING INEQUITIES IN PEDIATRIC ANAPHYLAXIS IN ITALY FROM THE SIAIP ‘PRIMARY AND SECONDARY PREVENTION OF ALLERGIC DISEASE COMMISSION’

Over recent years, the Italian Society of Pediatric Allergy and Immunology (SIAIP) has consistently promoted awareness, education, and policy dialogue on pediatric anaphylaxis through the organization of national congresses, dedicated scientific sessions, and thematic meetings focusing on prevention, emergency management, and health equity. Anaphylaxis represents a recurrent and central topic across SIAIP scientific activities, reflecting both its clinical relevance and the persistent gaps in preparedness, training, and access to life‐saving therapies. In parallel, SIAIP has actively supported educational initiatives and public awareness campaigns aimed at healthcare professionals, schools, families, and the wider community, in alignment with international efforts to reduce disparities in allergic disease outcomes.

In this context, the SIAIP Primary and Secondary Prevention of Allergic Diseases Commission proposes a structured, equity‐oriented action framework to translate current knowledge into implementable strategies. This framework identifies priority actions across different time horizons and levels of intervention, aiming to reduce disparities in prevention, recognition, and management of pediatric anaphylaxis in Italy.

The proposed SIAIP model is structured across short‐, medium‐, and long‐term priorities, reflecting a progressive implementation pathway that integrates clinical practice, public health policy, and healthcare system organization. This temporal stratification allows the identification of immediately actionable interventions alongside system‐level and structural strategies required to achieve sustainable reductions in inequities.

### 5.1 Short‐term priorities (0–12 months): Strengthening preparedness and standardization of care

Short‐term actions focus on rapidly implementable, high‐impact interventions aimed at addressing critical gaps in emergency preparedness and ensuring consistency in clinical practice.

Key priorities include the systematic delivery of standardized, hands‐on training in EAI use at every clinical encounter for children at risk of anaphylaxis, with mandatory periodic reinforcement to maintain competency. In parallel, pediatricians/clinicians should adopt a uniform prescribing approach, ensuring that all children are equipped with two EAIs, in line with international best practices.

To enhance knowledge and reduce variability in management, the development and nationwide dissemination of standardized, evidence‐based educational materials tailored to families, school personnel, and healthcare providers is essential. Furthermore, the introduction of minimum, nationally endorsed training requirements for caregivers and school staff represents a critical step toward improving early recognition and timely treatment of anaphylaxis in community settings.

Collectively, these interventions are low‐cost, scalable, and capable of producing immediate improvements in the appropriate use of epinephrine and overall emergency readiness. Moreover, these measures may also contribute to mitigating the impact of reimbursement‐related barriers by promoting appropriate prescribing practices and optimizing the use of available resources.

### 5.2 Medium‐term priorities (1–3 years): Reducing structural and geographic inequities

Medium‐term priorities focus on addressing structural barriers that contribute to inequitable access to care across regions, with particular emphasis on reimbursement policies and healthcare organization. In the Italian context, this need is supported by recent national evidence demonstrating significant regional variability in the availability and reimbursement of EAIs in both pediatric and adult populations, with differences in the number of devices provided and eligibility criteria.^52^

A central objective is the harmonization of EAI availability and reimbursement across all Italian regions. Current regional variability in reimbursement criteria, including differences in the number of devices provided and in eligibility conditions, may lead to inconsistent access pathways and unequal preparedness among pediatric patients. Aligning reimbursement policies with international recommendations, ensuring the provision of two EAIs for all at‐risk children, should be considered a national priority.

Moreover, the implementation of stock epinephrine programs in schools, supported by national or regional legislation, is essential to guarantee timely treatment in educational settings.

Additional priorities include the expansion of telemedicine services and the development of mobile and community‐based outreach programs to improve access in rural or underserved areas. Strengthening integration between primary care providers, emergency services, and allergy specialists is also crucial to ensure continuity of care and reduce fragmentation within the healthcare system.

### 5.3 Long‐term priorities (3–5 years): Building sustainable and equity‐oriented health systems

Long‐term strategies focus on structural reforms aimed at ensuring sustainability, accountability, and continuous quality improvement.

In addition to the creation of a national registry for pediatric anaphylaxis and the development of standardized monitoring indicators, long‐term strategies should include the implementation of national frameworks to periodically review and harmonize reimbursement policies across regions. This would ensure that access to EAIs is consistently aligned with clinical needs rather than regional administrative criteria.

Regular audits and benchmarking processes should incorporate equity‐focused indicators, including regional differences in EAI access and prescription patterns, enabling continuous monitoring and targeted policy adjustments.

**FIGURE 2:**
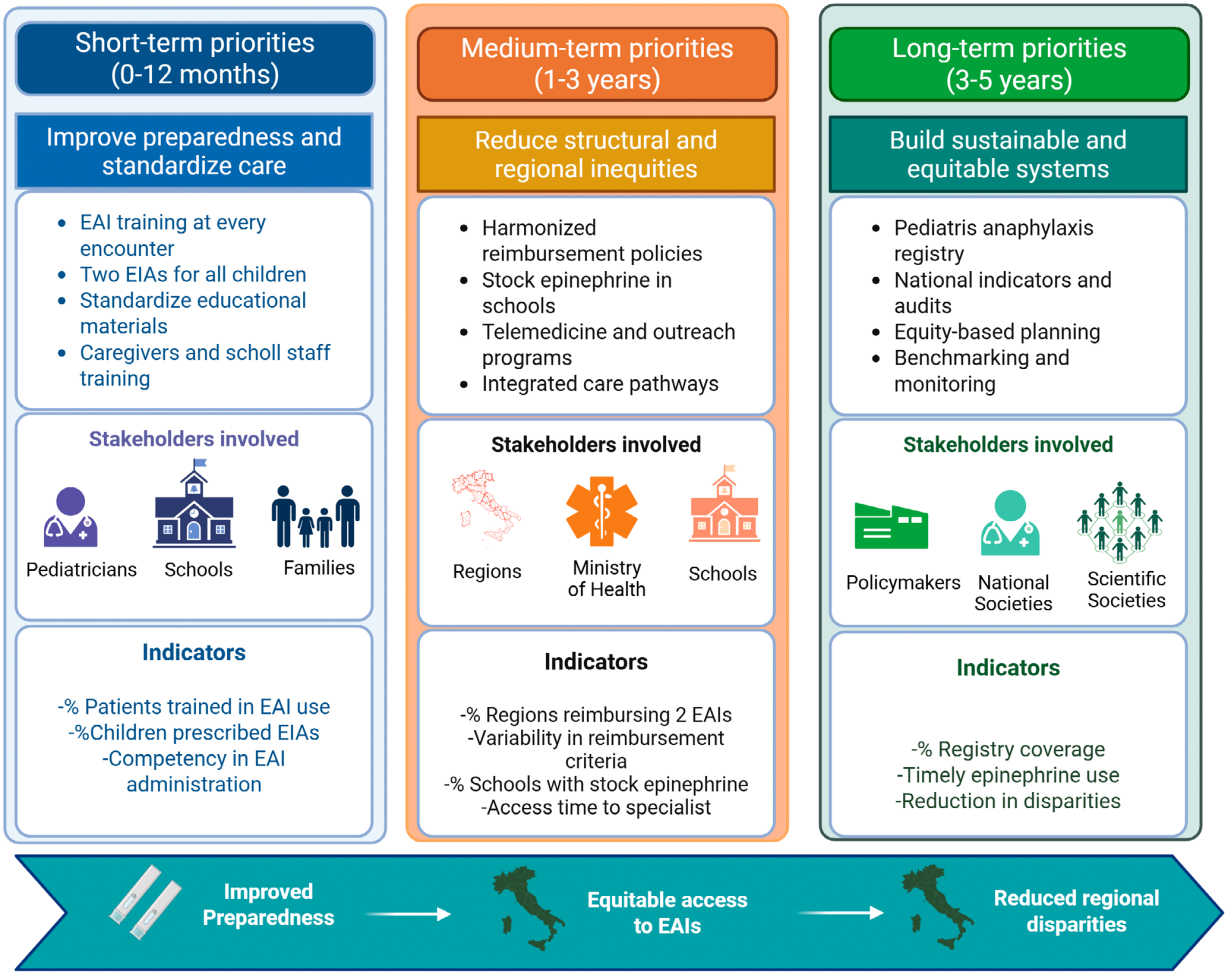
SIAIP Action Plan for reducing inequities in pediatric anaphylaxis in Italy. The figure illustrates a structured roadmap across short‐, medium‐, and long‐term priorities, integrating clinical, policy, and system‐level interventions. Key actions, stakeholders, and indicators are aligned to improve preparedness, ensure equitable access to EAIs, and reduce regional disparities. Created with BioRender.com.

Together, these measures provide the foundation for a learning healthcare system capable of continuously adapting to emerging evidence and reducing regional and socioeconomic inequalities in pediatric anaphylaxis care (Table 2 and Figure 2).

**TABLE 2:** SIAIP Action Plan for Pediatric Anaphylaxis in Italy: Priority levels, implementation components, and evaluation metric.

Priority level	Strategic objectives	Key actions	Stakeholders involved	Implementation barriers	Monitoring indicators
Short‐term (0–12 months)	Improve emergency preparedness and standardize clinical and community management	Standardized, repeated hands‐on EAI training at every clinical encounter Systematic prescription of two EAIs for all at‐risk children Development and national dissemination of evidence‐based educational materials Minimum standardized training requirements for caregivers and school personnel	Pediatricians, allergists, general practitioners, school staff, families, scientific societies	Time constraints in clinical practice; variability in training delivery; caregiver anxiety; lack of standardized educational tools	Proportion of patients receiving EAI training Proportion of children prescribed two EAIs Competency rates in correct EAI use Coverage of trained school personnel
Medium‐term (1–3 years)	Reduce geographic and structural inequities in access to care and treatment	Harmonization of EAI availability and reimbursement policies (number of devices and eligibility criteria) Implementation of stock epinephrine programs in schools Expansion of telemedicine services Development of mobile and community‐based outreach programs Strengthening integration between primary care, emergency services, and specialists	Regional health authorities, Ministry of Health, school systems, healthcare networks, policymakers	Regional heterogeneity in healthcare organization; funding limitations; −regulatory and legal barriers; infrastructure gaps	Proportion of regions reimbursing ≥2 EAIs per pediatric patient Variability in regional reimbursement criteria (number of devices and eligibility conditions) Proportion of pediatric patients eligible vs. not eligible for full reimbursement Proportion of schools equipped with stock epinephrine Access time to allergy specialist care
Long‐term (3–5 years)	Establish sustainable, equity‐oriented healthcare systems and continuous monitoring	Creation of a national pediatric anaphylaxis registry Development of standardized national indicators Implementation of periodic audits and benchmarking Integration of equity metrics into national healthcare planning	National health institutions, SIAIP, policymakers, public health agencies, data governance bodies	Data integration challenges; variability in regional data systems; long implementation timelines; governance complexity	National registry coverage Rates of timely epinephrine administration Reduction in regional disparities Trends in morbidity and healthcare utilization

## AUTHOR CONTRIBUTIONS

**Francesca Mori:** Supervision. **Leonardo Tomei:** Supervision. **Antonio Andrea Senatore:** Supervision. **Angela Klain:** Data curation; writing – original draft; resources. **Carolina Grella:** Writing – original draft; data curation; resources. **Francesca Galletta:** Supervision. **Sara Manti:** Supervision. **Amelia Licari:** Supervision. **Cristiana Indolfi:** Conceptualization; methodology; writing – review and editing. **Gian Luigi Marseglia:** Supervision. **Michele Miraglia del Giudice:** Supervision.

## FUNDING INFORMATION

The authors have nothing to report.

## CONFLICT OF INTEREST STATEMENT

The authors declare that they have no competing interests.

## Data Availability

Data sharing not applicable to this article as no datasets were generated or analysed during the current study.
